# Influence of Cardiac Decentralization on Cardioprotection

**DOI:** 10.1371/journal.pone.0079190

**Published:** 2013-11-13

**Authors:** John G. Kingma, Denys Simard, Pierre Voisine, Jacques R. Rouleau

**Affiliations:** 1 Département de médecine, Pavillon Ferdinand-Vandry, 1050, avenue de la Médecine, Université Laval, Québec (Québec), Canada; 2 Research Center, Institut universitaire de cardiologie et de pneumologie de Québec, 2725, chemin Sainte-Foy, Québec, Qc, Canada; 3 Département de chirurgie, Pavillon Ferdinand-Vandry, 1050, avenue de la Médecine, Université Laval, Québec (Québec), Canada; University of Louisville, United States of America

## Abstract

The role of cardiac nerves on development of myocardial tissue injury after acute coronary occlusion remains controversial. We investigated whether acute cardiac decentralization (surgical) modulates coronary flow reserve and myocardial protection in preconditioned dogs subject to ischemia-reperfusion. Experiments were conducted on four groups of anesthetised, open-chest dogs (n = 32): 1- controls (CTR, intact cardiac nerves), 2- ischemic preconditioning (PC; 4 cycles of 5-min IR), 3- cardiac decentralization (CD) and 4- CD+PC; all dogs underwent 60-min coronary occlusion and 180-min reperfusion. Coronary blood flow and reactive hyperemic responses were assessed using a blood volume flow probe. Infarct size (tetrazolium staining) was related to anatomic area at risk and coronary collateral blood flow (microspheres) in the anatomic area at risk. Post-ischemic reactive hyperemia and repayment-to-debt ratio responses were significantly reduced for all experimental groups; however, arterial perfusion pressure was not affected. Infarct size was reduced in CD dogs (18.6±4.3; p = 0.001, data are mean±1SD) compared to 25.2±5.5% in CTR dogs and was less in PC dogs as expected (13.5±3.2 vs. 25.2±5.5%; p = 0.001); after acute CD, PC protection was conserved (11.6±3.4 vs. 18.6±4.3%; p = 0.02). In conclusion, our findings provide strong evidence that myocardial protection against ischemic injury can be preserved independent of extrinsic cardiac nerve inputs.

## Introduction

Intrathoracic ganglia on the heart and their interconnections coordinate with central neurons in the spinal cord, brain stem and supraspinal central neuronal regions and modulate autonomic control of heart function. [1] As such, control of heart function involves a hierarchy of neurons located within: central command (top level), intrathoracic extracardiac ganglia (mid level) and intrinsic cardiac ganglia (bottom level). [2], [3] Alterations in neuronal input at any level could affect overall cardiac control; [4]–[6] however, despite altered connectivity neurons within the intrinsic cardiac nervous system can generate spontaneous activity and regulate regional cardiac function reflexively [7].

Within the left ventricle sympathetic and parasympathetic nerves are localized near cardiac myocytes to permit rapid crosstalk [8]– loss of this crosstalk could influence myocyte responses to ischemia. The role of cardiac nerves on development of ischemic injury remains controversial. Regional myocardial ischemia activates the autonomic nervous system; excessive stimulation produces electrical instability and increased incidence of atrial and ventricular arrhythmias. [9], [10] The latter also alters the myocardial oxygen supply demand relationship to produce greater post-ischemic myocardial injury. Development of post-ischemic tissue injury has been studied in isolated heart and *in situ* experimental preparations where extracardiac nerves have been sectioned. While many studies use the term ‘cardiac denervation’ to describe their experimental preparation after surgical ablation of extracardiac inputs use of ‘cardiac decentralization’ may be a more accurate descriptor of the animal model as mid and bottom levels of the neuronal hierarchy appear to be intact. In large animal studies, disruption of the extracardiac nervous system exerts either protective [11] or adverse effects [12], [13] on infarct size after ischemic injury. Huang *et al* reported increased myocardial stunning and patchy necrosis in cardiac denervated animals and suggested that the absence of cardiac nerves impaired recovery of cardiac function. [14] Delayed, but not first window, preconditioning in a cardiac denervated porcine model requires intact cardiac nerves. [15] These findings document a critical role for cardiac nerves in development of ischemic injury and eventual recovery thereof. The present study in an *in situ* canine preparation examined the effect of cardiac decentralization on post-ischemic coronary vascular reserve and development of acute ischemic injury. We hypothesized that a loss of central command inputs (i.e. sympathetic and parasympathetic control) to the local neuronal hierarchy would diminish coronary vascular reserve and worsen myocyte necrosis.

## Methods

Adult mongrel dogs of either sex (20–25 Kg) were used for these studies. Dogs were treated in compliance with the *Guide for the Care and Use of Laboratory Animals* published by the US National Institutes of Health (NIH publication 85-23, revised 1996); Laval University is compliant with these guidelines (A5012-01). The experimental protocol was approved by the Laval University Animal Ethics Committee.

### Surgical Preparation

Dogs were pre-medicated with acepromazine maleate (Atravet, 0.5 mg/Kg IM); anesthesia was induced with sodium pentobarbital (30 mg/Kg IV) and maintained with hourly administrations of 5 mg/Kg IV. After endotracheal intubation, dogs were ventilated with oxygen-enriched room air; respiratory rate and tidal volume were adjusted to maintain blood gases within physiological values. Butorphenol (0.2 mg/Kg IM) was administered for analgesia. Normothermia (38±1°C) was maintained with a water-jacketed Micro-Temp heating blanket (Zimmer, Dover, OH); temperature was continuously monitored with a thermal probe in the trachea and saline was given IV (250 mL/h) to replace fluid loss.

In the supine position, vascular introducer sheaths (8 Fr, Terumo Medical Corp., USA) were placed in the left and right femoral arteries; a triple-lumen central venous catheter (7 Fr, Arrow-Howes™, Arrow Intl. Inc., Reading, PA) was placed in the right femoral vein for administration of drugs and fluids. In the left lateral position the heart was exposed by thoracotomy. Extracardiac nerves were carefully dissected and excised; the stellate ganglia, ansae subclaviae and vagus nerves were targeted bilaterally. [16], [17] Completeness of cardiac decentralization prior to coronary occlusion and at the end of the experiment was verified by direct electrical stimulation and confirmed by the absence of change in heart rate and LV dynamics [13].

The heart was suspended in a pericardial cradle. The left anterior descending artery (LAD) was dissected distal to the first diagonal branch to allow positioning of a vascular clamp (for determination of coronary reactive hyperemia (RH) responses and coronary occlusion (CO)) and a blood volume flow probe (Transonic Systems, Ithaca, NY). Polyethylene catheters (7 Fr) were inserted into the internal thoracic artery (withdrawal of reference blood samples for microsphere studies) and left atrium (injection of microspheres). A 5 Fr micro-tipped pressure transducer (MPC500; Millar Instruments Inc., Houston, TX) was placed in the LV cavity through the apex to measure LV pressure and its first derivative; a 7 Fr Pigtail catheter was advanced to the aortic root via the left femoral artery to measure arterial pressures. After all catheters were positioned dogs were given heparin sodium (25 IU/Kg, IV) and allowed to stabilize for 30-min prior to data collection.

Left atrial and ascending aorta catheters were connected to Statham P23Db strain gauge manometers; zero was set at mid-chest level. The Millar micromanometer transducer was cross-calibrated with systolic aortic and diastolic left atrial pressures. Coronary blood flow was measured using a volume flow meter (Transonic Systems, Ithaca, NY). All data were continuously recorded and stored on computer hard drive for later analysis using AxoScope acquisition software. These parameters include heart rate, LV and aortic systolic/diastolic pressures, phasic and mean coronary blood flow. Rate-pressure product (RPP) was used as an indicator of myocardial oxygen demand and has been reported to correlate with myocardial oxygen consumption [18].

### Experimental Protocol

**Figure 1:**
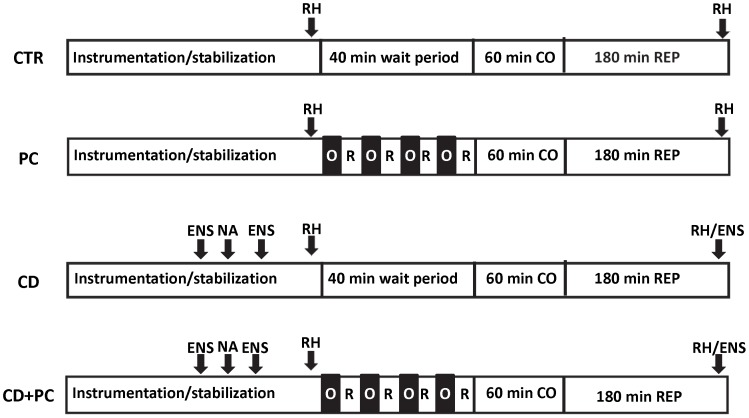
Schematic illustration of the experimental protocols. All dogs underwent 60-min acute CO and REP180. In CTR and CD dogs a 40-min wait period was allowed to compensate for time required for PC (4 cycles of 5-min CO/5-min reperfusion). The time at which reactive hyperemia (RH), nerve ablation (NA) and electrical nerve stimulation (ENS; outlined in Methods) was done is shown.

The experimental design is depicted schematically in Fig. 1. Dogs were randomly assigned to one of four groups; controls (CTR) and cardiac decentralized (CD) dogs underwent a 40-min wait period (equivalent to time of ischemic preconditioning (PC) protocol). For PC we used the classical model initially reported by Murry et al. [19] PC and CD+PC dogs were subjected to 4 cycles of 5-min LAD occlusion and 5-min reperfusion. All dogs underwent 60-min acute CO and 180-min reperfusion (REP180).

RH responses within the ischemic stress zone (i.e. LAD vascular bed) were obtained to assess the maximal vasodilator capacity of this vascular bed under stable conditions prior to PC and at the end of the ischemia-reperfusion protocol; a 20-sec acute, regional CO was used as this degree of ischemia produces maximal dilatation [20] rather than adenosine since the latter can act as a preconditioning mimetic [21].

All dogs were given lidocaine (5 mg/Kg, IV) at 55-min CO to suppress ventricular arrhythmias. Hearts that developed ventricular fibrillation or sustained ventricular tachycardia were cardioverted (1 J/Kg) using internal paddles.

### Coronary Collateral Blood Flow Measurements

Transmural myocardial blood flow was measured at 30-min CO using neutron activated microspheres (±15 µm; BioPAL Inc., Worcester, MA, U.S.A.) as previously described [22].

### Area at Risk and Infarct Size

At the end of each study, the LAD was re-ligated at the original site of occlusion; the area at risk (AAR) was outlined by perfusion of the coronary ostium with Monastral blue dye. Under deep pentobarbital anesthesia, cardiac arrest was induced by intra-atrial injection of saturated potassium chloride. A 1.5% solution of warmed (37°C) 2,3,5-triphenyltetrazolium chloride was infused into the ischemic region via a cannula in the LAD (distal to the snare occluder) over 30-min. The heart was rapidly excised, rinsed in saline and fixed in 10% buffered formaldehyde. The LV was cut from apex to base and the outline of each slice, the necrotic area (AN) and the AAR were traced onto acetates. The LV area, AAR and AN were determined using a digitizing tablet (Summagraphics II Plus) interfaced with a personal computer and analyzed with Sigma Scan software (SPSS Inc., CA, U.S.A.). Results are expressed as the AAR and the AN indexed to AAR (%). Tissue samples from the mid-region of the LV within the AAR and from the non-ischemic LV were further subdivided into endocardial and epicardial pieces for blood flow (i.e., coronary collateral flow) analyses; blood and tissue samples were dried for 48 h at 50°C and sent to a core processing facility (BioPAL Inc., Worcester, MA); [22] blood flow is expressed as mL/min/g.

### Data and Statistical Analyses

Differences in cardiac hemodynamics and regional blood flow were determined by ANOVA and multiple comparisons were performed using the Student-Newman-Keuls multiple range test. A probability (p) level of ≤0.05 was considered statistically significant; normality and variance assumptions were fulfilled. The influence of ventricular tachycardia/fibrillation on survival after the combined ischemia-reperfusion insult was determined using the Fisher Exact test and Chi-square analysis; all statistical analyses were carried out using SAS software (SAS Institute Inc., Cary, NC, U.S.A.). Sample size determination for these studies was based on the provision of a 90% power to detect, at a p≤0.05 significance level, a minimum 20 percent reduction/augmentation (expected standard deviation of ±8%) in infarct size.

RH responses were measured from strip chart recordings, baseline (Q_base_) and peak (Q_peak_) flow, flow debt and repayment volumes were determined as previously described; [20], [23] data are expressed as repayment-to-debt ratio. Coronary vascular conductance (CVC) at baseline and at peak flow during RH responses was calculated as the quotient of diastolic coronary blood flow and aortic pressure.

Development of tissue necrosis in the anesthetized canine is dependent on cardiac hemodynamics, AAR [18] and density of native collaterals; [24] all of these variables were considered in the statistical analyses. Coronary collateral blood flow within the ischemic zone (microspheres) was assessed at 30-min CO and infarct size was normalized to coronary collateral blood flow. Treatment effects of variables measured once (i.e., AN, AAR) were analyzed by ANOVA. [18] An analysis of covariance (ANCOVA) was also done to assess differences between experimental groups when variability due to coronary collateral flow (independent variable) was considered. The regression between infarct size and coronary collateral blood flow within the AAR was determined by a linear least-squares fit method. All analyses were conducted using the statistical package SAS, version 9.2 (SAS Institute Inc, Cary, NC).

## Results

Thirty-two dogs (n = 8 per group) were randomly allocated to the study groups and all completed the experimental protocol. Incidence of ventricular tachycardia/fibrillation during ischemia/reperfusion (0/8 CTR; 2/8 PC; 1/8 CD; 1/8 CD+PC) was not statistically different. Arterial blood gas and hematocrit values (data not shown) were all within physiological levels.

Cardiac hemodynamic data are summarized in Table 1. Heart rate (HR), LV systolic pressure (LVPS) and mean arterial pressure (MAP) were all lower in CD dogs. LV dP/dt+ and dP/dt− which is used to assess LV contractility was significantly lower in CD dogs; CO produced a significant drop in dP/dt+ in all dogs. During reperfusion LV dP/dt+ values were consistently lower.

**Table 1:** Summary of cardiac hemodynamic data.

Group	Intervention	HR		LVPS	MAP	RPP	dP/dt+	dP/dt-
***CTR***								
	**Baseline**		154±19	111±14^a^	93±14	17.1±3.1	1797±440^a^	1382±136
	**30-min CO**		144±18	110±9^a^	92±9	15.9±2.3	1575±294^b^	1319±129
	**30-min REP**		141±17	106±11^b^	89±10	14.9±2.6	1364±174^b,c^	1253±152
	**180-min REP**		147±10	104±10^b^	89±10	15.3±2.1	1259±190^c^	1229±147
***PC***								
	**Baseline**		141±29	115±12^a^	98±10	16.2±3.7	1820±564^a^	1335±196
	**30-min CO**		141±30	106±9^b^	88±8	14.9±3.0	1560±326^b^	1287±239
	**30-min REP**		143±29	104±10^b^	86±11	14.8±3.0	1470±309^b,c^	1301±224
	**180-min REP**		150±27	102±10^b^	87±10	15.4±3.5	1402±217^c^	1364±233
***CD***								
	**Baseline**		115±19	102±8^a^	82±8	11.7±2.4	1360±264^a^	1131±236
	**30-min CO**		112±15	96±8^b^	76±1	10.7±2.1	1201±195^b^	958±152
	**30-min REP**		120±17	100±7^b^	82±9	12.0±1.7	1217±152^b,c^	1114±158
	**180-min REP**		119±11	98±20^b^	83±19	11.7±2.9	1021±313^c^	1044±282
***CD+PC***								
	**Baseline**		102±16	113±15^a^	93±13	11.6±2.5	1284±197^a^	1141±180
	**30-min CO**		103±14	102±12^b^	84±13	10.6±2.6	1143±184^b^	971±269
	**30-min REP**		108±15	104±10^b^	85±11	11.2±2.5	1147±159^b,c^	1081±178
	**180-min REP**		115±20	99±10^b^	83±9	11.5±2.8	1049±144^c^	1112±187
**P(Groups)**			0.001	0.010	0.003	0.001	0.001	0.001
**P(Inter)**			NS	0.007	NS	NS	0.001	NS
P(Groups*Inter)			NS	NS	NS	NS	NS	NS

Data are means±1SD (n = 8 per group); CO, coronary occlusion; REP, reperfusion. HR: heart rate (beats/min); LVPS (mmHg): LV systolic pressure; MAP (mmHg): mean arterial pressure; RPP (beats/min X mmHg/1000): rate-pressure product; LV dP/dt+, dP/dt− (mmHg/s): index of LV function during ventricular contraction, relaxation; p value using ANOVA with degree of freedom, df: 15,111. Multiple comparisons between experimental groups and within interventions were performed using ANOVA with the Student’s-Newman-Keuls multiple range test; means with similar letters are not statistically different.

**Figure 2:**
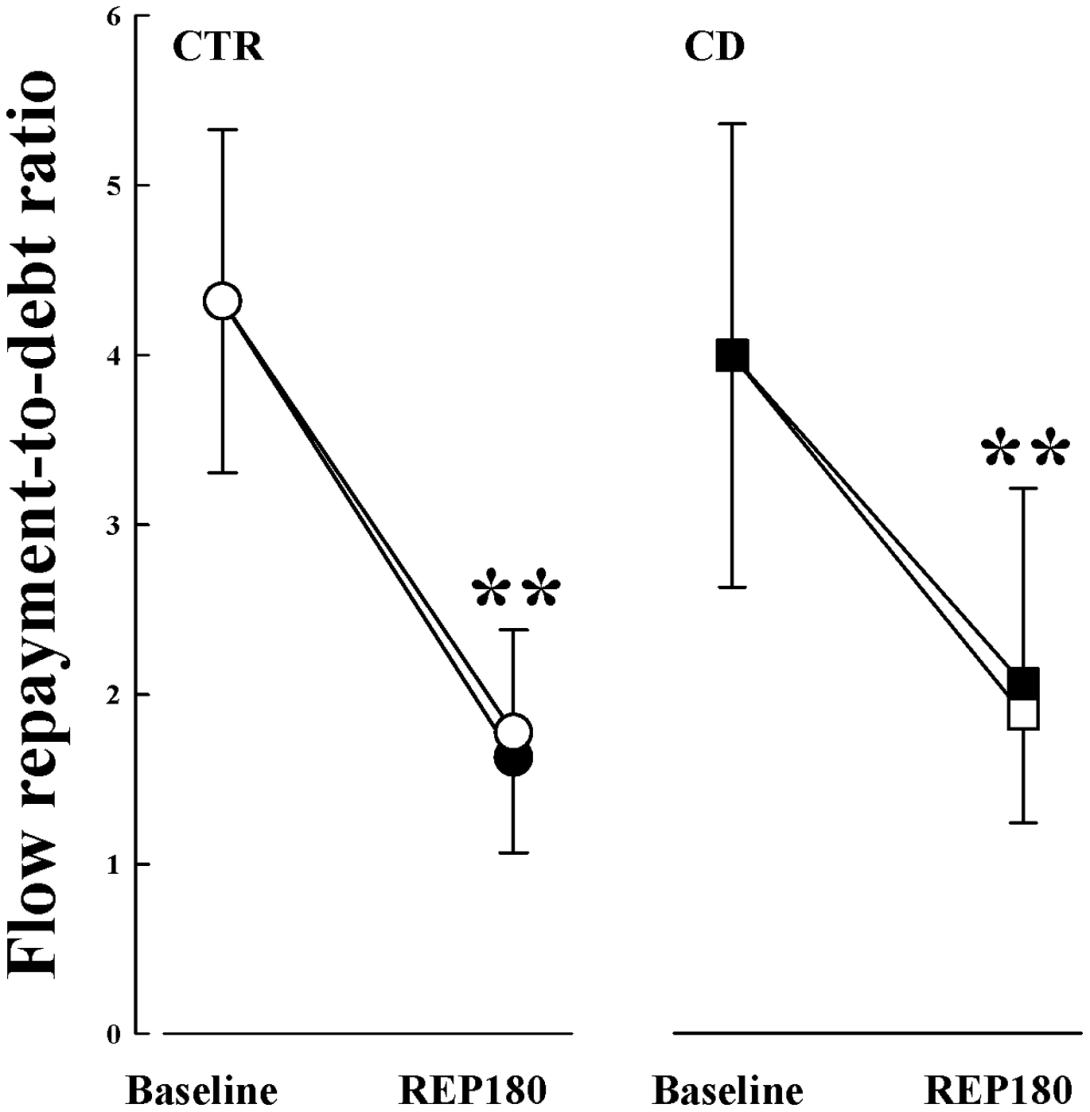
Repayment-to-debt ratios of coronary blood flow during RH in CTR (open circles), PC closed circles), CD (open squares) and CD+PC (closed squares) before CO (i.e. baseline) and at REP180. Data are means±1SD; **p≤0.05 vs. before ischemia.

Changes in coronary RH responses (used to assess coronary flow reserve) produced by ischemia-reperfusion injury are reported in Table 2. After REP180, Q_base_ was significantly reduced in all groups and Q_peak_ decreased almost 50 percent less for each group; as such, 60-min CO resulted in an overall loss of coronary flow reserve (cf. Q_peak_/Q_base_) regardless of cardiac nerve status. While no change in CVC_base_ was detected at REP180, CVC_peak_ was significantly less in all experimental groups. Coronary blood flow repayment-to-debt ratio decreased markedly following ischemia-reperfusion in CTR and CD dogs even though arterial perfusion pressures remained stable (cf. Figure 2); no change was observed with PC pretreatment.

**Table 2:** Summary of coronary reactive hyperemia responses.

Groups	PAoD	Q_base_	Q_peak_	Q_peak_/Q_base_	CVC_base_	CVC_peak_
	**Baseline**					
***CTR***	85±15	37±16	155±52	4.4±0.1	0.34±0.15	1.43±0.27
***PC***	91±10	39±11	180±57	4.6±1.2	0.44±0.16	2.12±0.91
***CD***	73±10	36±10	151±55	4.2±1.2	0.39±0.12	1.69±0.60
***CD+PC***	84±12	31±11	137±36	4.6±0.8	0.33±0.14	1.60±0.42
	**REP180**					
***CTR***	83±10	32±10	85±22	2.7±0.5	0.38±0.18	1.00±0.32
***PC***	80±11	28±14	89±55	3.1±0.6	0.31±0.19	0.97±0.68
***CD***	76±20	26±10	77±28	3.0±0.4	0.27±0.14	0.98±0.46
***CD+PC***	76±9	24±13	77±38	3.7±1.6	0.25±0.10	0.85±0.43
**P (Groups)**	0.002	NS	NS	NS	NS	NS
**P (Inter)**	NS	0.010	0.001	0.001	NS	0.001
P (Groups* **Inter)**	NS	NS	NS	NS	NS	NS

Data are means ±1SD (n = 8 dogs/group); Baseline: before ischemia-reperfusion; REP, reperfusion; PAoD: diastolic aortic pressure; Q_base_: baseline blood flow (mL/min); Q_peak_: peak blood flow (mL/min) after 20-sec LAD occlusion; CVC_base_, CVC_peak_: coronary vascular conductance at baseline and at peak flow during RH responses.

**Figure 3:**
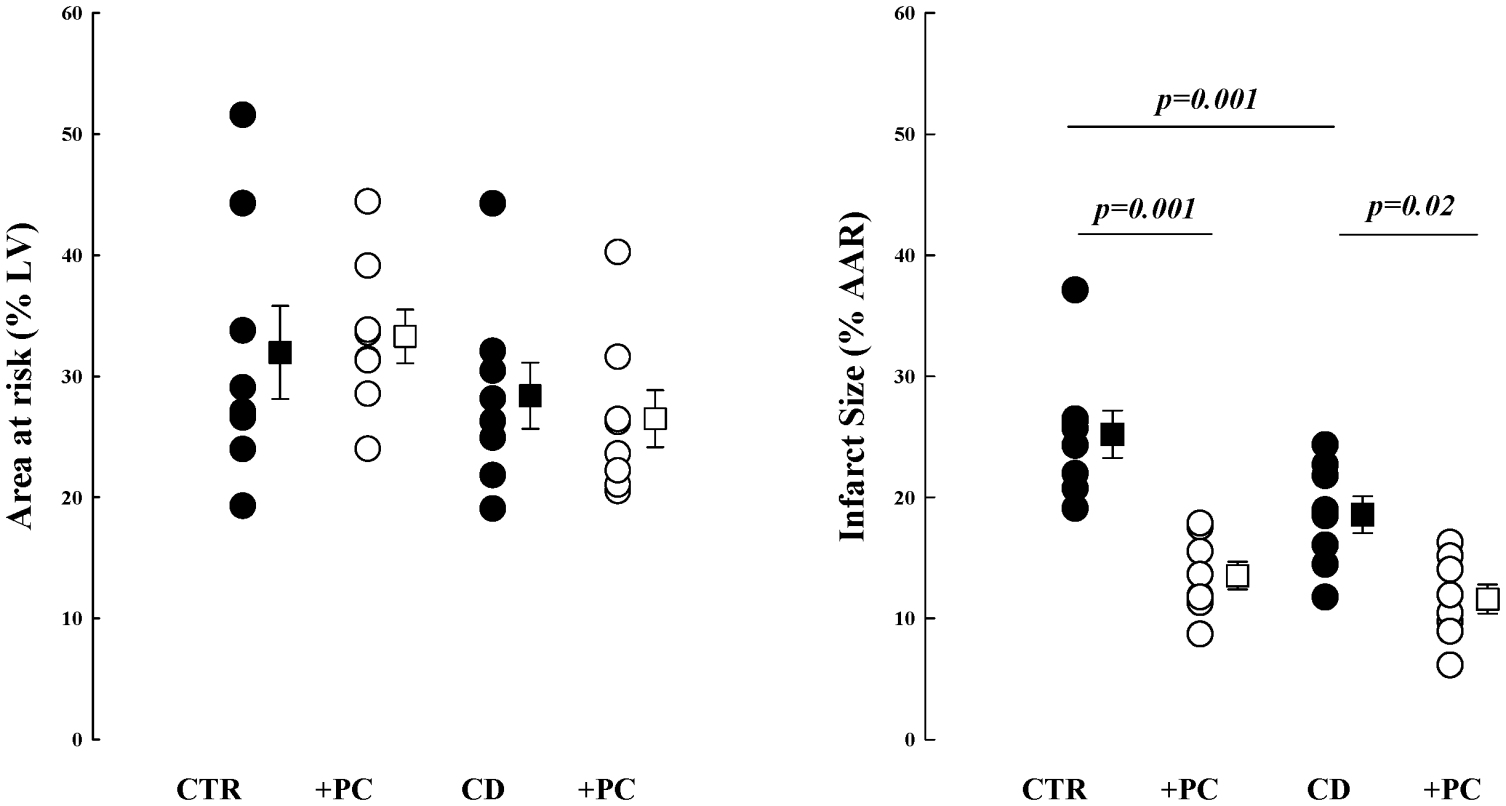
Area at risk (% LV area; left panel) and myocardial infarct size (% AAR; right panel) are shown for all study groups. Data are means±SEM; differences between groups were established by ANOVA with the Student’s-Newman-Keuls multiple range test.

The AAR (% LV area) was similar for all experimental groups (CTR, 32±11; PC, 33±6; CD, 28±8; CD+PC, 27±7) as shown in Figure 3. As expected, infarct size (%AAR) was significantly decreased in PC (13.5±3.2%, mean±1SD, p = 0.001) compared to CTR (25.2±5.5%) dogs. Cardiac decentralization resulted in smaller infarcts compared to CTR dogs (18.6±4.3 vs. 25.2±5.5%; p = 0.001); infarct size was further reduced by PC in CD dogs (11.6±3.4%). The level of ischemia for each dog was similar as indicated by the level of coronary collateral blood flow within the AAR measured by microspheres at 30-min coronary occlusion (CTR, 0.06±0.02; PC, 0.07±0.03; CD, 0.07±0.02; CD+PC, 0.06±0.02 mL/min/g). Blood flow within non-ischemic myocardium at the same time point was: CTR, 1.51±0.34; PC, 1.13±0.19; CD, 1.25±0.21; CD+PC, 1.20±0.09 mL/min/g; p = NS).

**Figure 4:**
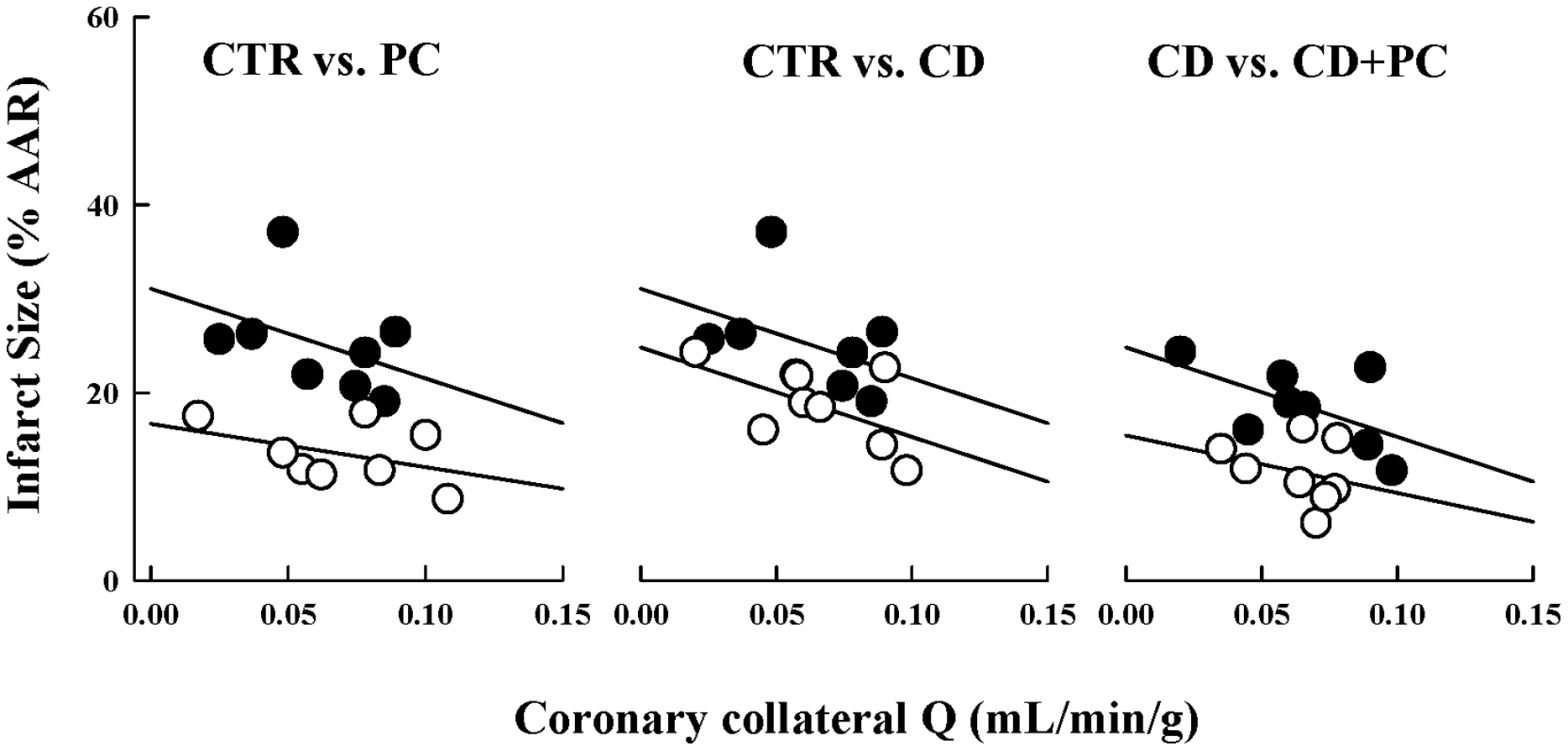
Infarct size (% AR) versus coronary collateral blood flow (mL/min/g). Left panel: CTR (closed symbols; y = 32.4−112.6X, r^2^ = 0.203) versus PC (open symbols; y = 11.6−32.9X, r^2^ = 0.055) dogs. Middle panel: CTR (closed symbols; y = 32.4−112.6X, r^2^ = 0.203) versus CD (open symbols; y = 24.9−96.4X, r^2^ = 0.327). Right panel: CD (closed symbols; y = 24.9−96.4X, r^2^ = 0.327) versus CD+PC (open symbols; y = 15.5−60.7X, r^2^ = 0.079) dogs. The downward shift of the regression indicates significant protection that occurs independently of changes in coronary collateral blood flow. Each point represents an individual dog.

Although direct comparisons of infarct size by ANOVA demonstrate reduced tissue injury by PC (or CD) this statistical method does not take into consideration the important influence of coronary collateral blood flow (independent covariant) within the AAR on development of tissue necrosis. In CTR dogs an inverse relationship between coronary collateral blood flow and infarct size was obtained (i.e., low regional blood flow results in larger infarcts). [19] The infarct size/coronary collateral blood flow relation was shifted downward for PC, CD and CD+PC groups as shown in Figure 4; slopes of the regressions were similar for all experimental groups. These results suggest significant cardioprotection independent of coronary collateral flow levels in PC, CD and CD+PC dogs.

## Discussion

Results of the present study show that a 60-min period of ischemia substantially reduces coronary vascular reserve; a similar impairment of this parameter was observed after acute ablation of all extracardiac neuronal inputs (i.e. cardiac decentralization). These effects were independent of pretreatment by cardiac conditioning. Myocardial necrosis was significantly reduced in CD dogs; a similar level of tissue protection was afforded by conditioning pretreatment independent of CD.

Coronary flow reserve can evaluate coronary microvessel function and provides well-established risk and prognostic variables. [25] Reduced cardiac contractile function post-ischemia further affects the relation between adequate oxygen supply and ventricular remodeling. [26] In the present study coronary flow within the ischemic stress zone after ischemia and reperfusion was significantly diminished in all experimental groups; peak flow values were also markedly lower and resulted in a marked lowering of coronary flow reserve. Interestingly, neither ischemic preconditioning nor cardiac decentralization attenuated the effect of ischemia on coronary flow reserve. These data are not consistent with earlier studies showing preserved coronary function by cardiac conditioning. [27]–[30] We expected to see improved coronary vessel function in PC and CD animals due to either upregulation of intracellular cytoprotective pathways or reduced metabolic demands, respectively. Furthermore, these data cannot explain the observed reduction in infarct size in either PC or CD animals; a close relation has been reported between infarct size and distribution of myocardial blood flow within the anatomic risk area [31].

In CD animals not subject to PC pretreatment infarct size was significantly smaller. While these findings are in agreement with those previously reported [11], [32] important differences exist with respect to the cardiac denervation and coronary occlusion protocols used. Furthermore, our findings contrast with those reported by Lavallee *et al* in dogs subject to total coronary occlusion. [13] Many different conditioning strategies either *in situ* in animals with intact cardiac nerves or in isolated buffer-perfused hearts have been reported in the literature to reduce myocardial injury and infarct size. [33] We report here that preconditioning mediated protection against ischemia-reperfusion injury was not abrogated by cardiac decentralization. Kudej *et al* also documented significant infarct size reduction by PC pretreatment after acute cardiac denervation (using surgical and chemical methods). [15] While they maintain that intact cardiac nerves are not essential for first window preconditioning we contend that the role functional neurons within the cardiac ganglia play in post-ischemic myocardial adaptation needs to be better defined. Indeed, sub-populations of neurons have even been shown to influence reflex cardiac functions even after their physical disconnection from central command [34], [35] due to maintained synaptic communication between intrinsic cardiac neurons [4].

Sympathetic dysinnervation secondary to myocardial infarction has been reported in animal and human studies. [36], [37] However, the injury threshold of cardiac neurons (sympathetic and parasympathetic) during ischemia-reperfusion remains unclear; sympathetic impairment might also exceed the area of decreased perfusion and myocyte necrosis. [38], [39] Ischemic stress stimulates release of autocoids (i.e. adenosine, bradykinin), nitric oxide and reactive oxygen species that trigger cellular signal transduction pathways; most of these compounds initiate responses in somata and axons of the mammalian intrinsic cardiac nervous system. [40] It is conceivable that intrinsic cardiac neurons and ischemic preconditioning share common pathways to stimulate survival of myocytes against ischemia; the intrinsic cardiac nervous system might even play an important role in mediating the benefits of preconditioning.

There are some limitations in our study. We used an open-chest, isoflurane-anesthetized canine experimental preparation. *A priori* consideration was not given to either surgical or anesthetic preconditioning since all animals were treated similarly. Additional risk factors that influence myocardial infarct size such as duration and depth (i.e., residual flow deficit in the ischemic zone) of ischemia and anatomic risk zone size were measured in the present studies and were uniform for all study groups. [41], [42] In this paper we use the term ‘decentralized’ rather than ‘denervated’ to describe the experimental model; earlier studies used surgical or chemical ablation methods, or a combination of both to create the so-called cardiac denervation model. Surgical ablation of intrapericardial (ventrolateral cardiac nerve and stripping tissue from pulmonary veins, pulmonary artery and superior vena cava) nerve inputs to the intrinsic cardiac nervous system does not specifically target intrinsic cardiac ganglionated plexuses and associated pericardial nerves. Since these ganglia are functional use of the term cardiac denervation may be erroneous. As such, while acute bilateral ablation of extracardiac nerves, as done in the present study, enabled disconnection from central command (i.e. top level) peripheral nerve networks still regulate cardiac function. Future studies could be directed towards evaluation of the role of specific intrinsic cardiac nervous system ganglionated plexuses on conditioning-mediated cardioprotection.

In conclusion, cardioprotection against ischemic injury is conserved by preconditioning even when the target tissues are disconnected from central command. These findings suggest that preconditioning and the intrinsic cardiac nervous system could share common pathways to delay development of myocyte necrosis. Results of the present studies could have implications for understanding physiopathology of ischemia-induced heart failure. Further studies are needed to determine whether neuromodulation of the intrinsic cardiac nervous system would be beneficial against ischemia-reperfusion.
